# HIV Shedding from Male Circumcision Wounds in HIV-Infected Men: A Prospective Cohort Study

**DOI:** 10.1371/journal.pmed.1001820

**Published:** 2015-04-28

**Authors:** Aaron A. R. Tobian, Godfrey Kigozi, Jordyn Manucci, Mary K. Grabowski, David Serwadda, Richard Musoke, Andrew D. Redd, Fred Nalugoda, Steven J. Reynolds, Nehemiah Kighoma, Oliver Laeyendecker, Justin Lessler, Ronald H. Gray, Thomas C. Quinn, Maria J. Wawer, the Rakai Health Sciences Program

**Affiliations:** 1 Department of Pathology, School of Medicine, Johns Hopkins University, Baltimore, Maryland, United States of America; 2 Rakai Health Sciences Program, Entebbe, Uganda; 3 Department of Medicine, School of Medicine, Johns Hopkins University, Baltimore, Maryland, United States of America; 4 Department of Epidemiology, Bloomberg School of Public Health, Johns Hopkins University, Baltimore, Maryland, United States of America; 5 Institute of Public Health, Makerere University, Kampala, Uganda; 6 Division of Intramural Research, National Institute of Allergy and Infectious Diseases, National Institutes of Health, Bethesda, Maryland, United States of America; University of Bern, SWITZERLAND

## Abstract

**Background:**

A randomized trial of voluntary medical male circumcision (MC) of HIV—infected men reported increased HIV transmission to female partners among men who resumed sexual intercourse prior to wound healing. We conducted a prospective observational study to assess penile HIV shedding after MC.

**Methods and Findings:**

HIV shedding was evaluated among 223 HIV—infected men (183 self—reported not receiving antiretroviral therapy [ART], 11 self—reported receiving ART and had a detectable plasma viral load [VL], and 29 self—reported receiving ART and had an undetectable plasma VL [<400 copies/ml]) in Rakai, Uganda, between June 2009 and April 2012. Preoperative and weekly penile lavages collected for 6 wk and then at 12 wk were tested for HIV shedding and VL using a real—time quantitative PCR assay. Unadjusted prevalence risk ratios (PRRs) and adjusted PRRs (adjPRRs) of HIV shedding were estimated using modified Poisson regression with robust variance. HIV shedding was detected in 9.3% (17/183) of men not on ART prior to surgery and 39.3% (72/183) of these men during the entire study. Relative to baseline, the proportion shedding was significantly increased after MC at 1 wk (PRR = 1.87, 95% CI = 1.12–3.14, p = 0.012), 2 wk (PRR = 3.16, 95% CI = 1.94–5.13, p < 0.001), and 3 wk (PRR = 1.98, 95% CI = 1.19–3.28, p = 0.008) after MC. However, compared to baseline, HIV shedding was decreased by 6 wk after MC (PRR = 0.27, 95% CI = 0.09–0.83, p = 0.023) and remained suppressed at 12 wk after MC (PRR = 0.19, 95% CI = 0.06–0.64, p = 0.008). Detectable HIV shedding from MC wounds occurred in more study visits among men with an HIV plasma VL > 50,000 copies/ml than among those with an HIV plasma VL < 400 copies/ml (adjPRR = 10.3, 95% CI = 4.25–24.90, p < 0.001). Detectable HIV shedding was less common in visits from men with healed MC wounds compared to visits from men without healed wounds (adjPRR = 0.12, 95% CI = 0.07–0.23, p < 0.001) and in visits from men on ART with undetectable plasma VL compared to men not on ART (PRR = 0.15, 95% CI = 0.05–0.43, p = 0.001). Among men with detectable penile HIV shedding, the median log_10_ HIV copies/milliliter of lavage fluid was significantly lower in men with ART—induced undetectable plasma VL (1.93, interquartile range [IQR] = 1.83–2.14) than in men not on ART (2.63, IQR = 2.28–3.22, p < 0.001). Limitations of this observational study include significant differences in baseline covariates, lack of confirmed receipt of ART for individuals who reported ART use, and lack of information on potential ART initiation during follow—up for those who were not on ART at enrollment.

**Conclusion:**

Penile HIV shedding is significantly reduced after healing of MC wounds. Lower plasma VL is associated with decreased frequency and quantity of HIV shedding from MC wounds. Starting ART prior to MC should be considered to reduce male-to-female HIV transmission risk. Research is needed to assess the time on ART required to decrease shedding, and the acceptability and feasibility of initiating ART at the time of MC.

## Abstract 2

In this prospective cohort study, Aaron Tobian and colleagues examine the associations between male circumcision wound healing, as well as plasma viral load, and HIV shedding from male circumcision wounds.

## Editors' Summary

**Background:**

About 35 million people are currently infected with HIV, the virus that causes AIDS by destroying immune system cells, and every year, 2 million more people become HIV-positive. Antiretroviral therapy (ART) can keep HIV in check, but there is no cure for AIDS. Consequently, prevention of HIV acquisition and transmission is an important component of efforts to control the AIDS epidemic. Because HIV is most often spread through unprotected sex with an infected partner, individuals can reduce their risk of becoming HIV-positive by abstaining from sex, by having only one or a few partners, and by using male or female condoms. In addition, three trials undertaken in sub-Saharan Africa a decade ago showed that male circumcision—the surgical removal of the foreskin, a loose fold of skin that covers the head of the penis—can halve the HIV acquisition rate in men. Thus, since 2007, the World Health Organization (WHO) has recommended voluntary medical male circumcision for individuals living in countries with high HIV prevalence as part of its HIV prevention strategy.

**Why Was This Study Done?:**

With the rollout of voluntary medical male circumcision programs, circumcision has become more normative (regarded as acceptable), and HIV-positive men are increasingly requesting circumcision because they want to avoid any stigma associated with being uncircumcised and because circumcision provides health benefits. WHO recommends that, although circumcision should not be promoted for HIV-positive men, voluntary circumcision programs should operate on HIV-positive men if they request circumcision. However, in a trial of circumcision of HIV-infected men, HIV transmission to their female partners increased if the couples had sexual intercourse before the circumcision wound had healed. Moreover, in studies of current male circumcision programs, two-thirds of married men and a third of all men reported that they resumed sexual intercourse before their circumcision wounds had healed. Thus, better understanding of how male circumcision increases HIV transmission to female partners is essential, and improved ways to prevent transmission in the post-surgical period are needed. Here, in a prospective observational study (an investigation that collects data over time from people undergoing a specific procedure), the researchers assess HIV shedding from the penis after circumcision.

**What Did the Researchers Do and Find?:**

The researchers evaluated penile HIV shedding among 223 HIV-infected men (183 men who self-reported not being on ART and 40 men who self-reported being on ART, 29 of whom had no detectable virus in their blood) living in Rakai, Uganda, by examining preoperative and postoperative penile lavage (wash) samples. Viral shedding was detected in 9.3% of the men not on ART before surgery and in 39.3% of these men during the entire study. Relative to baseline, a greater proportion of men shed virus at one, two, and three weeks after circumcision, but a lower proportion shed virus at six and twelve weeks after circumcision. HIV shedding was more frequent among men with a high amount of virus in their blood (a high viral load) than among men with a low viral load. Moreover, the frequency of HIV shedding was lower in visits from men with healed circumcision wounds than in visits from men with unhealed wounds, and in visits from men on ART with no detectable virus in their blood than in visits from men not on ART men. Finally, among men with detectable penile HIV shedding, men on ART with no detectable virus in their blood shed fewer copies of virus than men not on ART.

**What Do These Findings Mean?:**

The findings suggest that healed circumcision wounds are associated with reduced penile HIV shedding in HIV-positive men compared to unhealed circumcision wounds and HIV shedding prior to circumcision In addition, they suggest that a lower HIV viral load in the blood is associated with a decreased frequency and quantity of HIV shedding from circumcision wounds. Because this was an observational study, these findings cannot prove that healed wounds or reduced blood viral load actually caused reduced penile HIV shedding. Moreover, the accuracy of these findings may be affected by the lack of information on ART initiation during follow-up among men not initially on ART and by reliance on ART self-report. Nevertheless, these findings highlight the importance of counseling HIV-positive men undergoing circumcision to avoid sexual intercourse until their circumcision wound heals. In addition, these findings suggest that it might be possible to reduce HIV transmission among HIV-positive men immediately after circumcision by starting these individuals on ART before circumcision. Further research is needed to assess how long before circumcision ART should be initiated and to assess the acceptability and feasibility of initiating ART concurrent with circumcision.

**Additional Information:**

Please access these websites via the online version of this summary at http://dx.doi.org/10.1371/journal.pmed.1001820.

- Information is available from the US National Institute of Allergy and Infectious Diseases on HIV infection and AIDS
- NAM/aidsmap provides basic information about HIV/AIDS, summaries of recent research findings on HIV care and treatment, information on male circumcision for the prevention of HIV transmission, and personal stories about living with HIV/AIDS
- Information is available from Avert, an international AIDS charity on many aspects of HIV/AIDS, including information on HIV prevention, on voluntary medical male circumcision for HIV prevention, and on HIV/AIDS in sub-Saharan Africa; Avert also provides personal stories about living with HIV/AIDS
- The World Health Organization provides information on all aspects of HIV/AIDS (in several languages), including on voluntary medical male circumcision for HIV prevention
- The UNAIDS Fast-Track Strategy to End the AIDS Epidemic by 2030 provides up-to-date information about the AIDS epidemic and efforts to halt it
- The Clearinghouse on Male Circumcision for HIV Prevention provides up-to-date information and resources on male circumcision for HIV prevention

## Introduction

Three randomized trials demonstrated that voluntary medical male circumcision, hereafter referred to as male circumcision (MC), significantly decreases heterosexual HIV acquisition in men by 50%–60% [1–3]. MC of HIV-negative men also reduces herpes simplex virus type 2 (HSV-2) and human papillomavirus (HPV) infection among heterosexual men and provides benefits to female partners, including reduced prevalence of genital ulcer disease (GUD), bacterial vaginosis, *Trichomonas vaginalis*, and HPV [4–14]. MC may also reduce incident syphilis in both men and their female partners [15]. MC of HIV-infected men reduces GUD and HPV [16,17]. Observational studies showed that MC prior to puberty decreased HIV transmission [18,19]. However, MC of HIV-infected men in a randomized trial had no impact on HPV transmission [20], and it increased HIV transmission to men’s female partners among couples who engaged in sexual intercourse prior to wound healing, though not among couples who delayed resumption of sex [17].

As MC has become more normative, HIV-infected men have sought to become circumcised for multiple reasons, including because of perceived health benefits, because they think they are HIV-negative and are seeking protection against HIV, or to avoid stigma [21]. If they are denied MC through organized programs, some HIV-infected men are likely to seek it from unsafe sources, potentially resulting in post-surgical complications and increasing the risk of HIV transmission [17,22]. Given these concerns, the World Health Organization (WHO) recommends that, although MC should not be promoted for HIV-infected men, these men should not be denied the service if they request it [21].

Even with counseling of post-surgical sexual abstinence, 65% of married men and approximately 30% of all men report resuming sexual intercourse prior to wound healing [23,24]. A better understanding of how MC might increase HIV transmission to female partners and of possible prevention methods in the immediate post-surgical period is urgently needed. A study of HIV-infected men found increased HIV shedding from MC wounds in the first weeks after the procedure [25]. To our knowledge, the association of HIV shedding from MC wounds and plasma viral load (VL), and the potential impact of antiretroviral therapy (ART) on HIV shedding have not been assessed. Here we report the frequency and quantity of penile HIV shedding among HIV-infected men undergoing MC to assess the a priori hypotheses that penile HIV shedding increases immediately after surgery, shedding is associated with plasma VL, and ART suppresses penile HIV shedding.

## Methods

### Study Population and Design

A prospective study was conducted between June 2009 and April 2012 in Rakai, Uganda. Uncircumcised HIV-infected men aged ≥15 y who requested free MC services and had no contraindication to surgery were invited to participate. All participants provided written informed consent prior to enrollment. All HIV-infected men were referred for HIV care. HIV-negative men were enrolled in a concurrent parallel study of MC wound healing to avoid stigmatization of the HIV-infected participants.

All men were offered the standard health services recommended by WHO prior to MC. These services included (1) free voluntary HIV counseling and testing, though acceptance was not a prerequisite for MC, (2) education on HIV prevention, risks and benefits of MC, wound care, and the need to abstain from intercourse until complete wound healing was certified, and (3) treatment of symptomatic sexually transmitted infections. Men were clinically assessed for contraindications to surgery (e.g., hypospadiasis, genital infections, hemoglobin <8.0 g/dl) by trained medical or clinical officers. Conditions such as urethral discharge or GUD were treated and resolved prior to MC.

At enrollment, information on sociodemographics, self-reported current ART use, and sexual behaviors was collected using structured questionnaires. Venous blood for HIV serology, plasma VL testing, and CD4 count was collected prior to surgery. MCs were conducted by trained clinical officers (96%) and medical officers (4%) by excising the foreskin using the dorsal slit method under aseptic conditions. All participants were followed weekly for 6 wk and then at 8 and 12 wk. Information on sexual behaviors and blood for plasma VL testing were obtained at each follow-up visit. Wound healing was assessed via direct observation starting at the 3-wk post-operative visit by clinical officers. Certified complete wound healing was defined as an intact scar with no scabs, sutures, or stitch sinuses.

Lavage samples to assess HIV viral shedding at the coronal sulcus prior to MC and at the MC surgical site were also collected. The lavage involved pouring 5 ml of phosphate buffered saline (pH 7.2) over the coronal sulcus or wound site. The lavage fluid was collected into a sterile cup, aliquoted in 1-ml vials, and stored at −80°C until assay, as described previously [26]. Lavage samples were collected just prior to surgery, intra-operatively immediately after suturing but prior to the routine end-of-surgery wash with normal saline, at 7 d post-MC following removal of the Sofra-Tulle dressing, then weekly during the follow-up visits. Samples at 5 and 8 wk were not evaluated since preliminary data showed essentially no HIV shedding from MC wounds from 5 wk post-MC onwards.

### HIV and CD4 Cell Count Testing

HIV status was determined by two enzyme immunoassays: the Murex HIV 1.2.0 followed by Vironostika HIV-1/2 Plus O (Organon Teknika). Discordant and indeterminate samples were subjected to BioMérieux Vitek Western blot confirmation, and to reverse transcription PCR (RT-PCR) when Western blot results were indeterminate. Determination of CD4+ T cell count at baseline used a three-color FACSCalibur (Becton-Dickinson).

### Plasma and Lavage HIV Viral Load Testing

Plasma HIV-1 RNA levels (VLs) were determined by an RT-PCR assay (Abbott Laboratories). Men were considered to have an undetectable plasma VL if they had <400 copies/ml at enrollment.

MC lavage samples were assessed using a modified version of the Abbott Laboratories RT-PCR assay with a detection limit of 40 copies/ml. The manufacturer’s instructions were followed with only four exceptions: (1) a total nucleic acid extraction was performed, (2) calibrators were included to perform the assay as a quantitative test, (3) microparticles were increased from 40 μl to 80 μl to increase analytical sensitivity, and (4) an extended drying step (15 min total) was performed to ensure that no ethanol or beads were in the final product.

### Statistical Analysis

As described in the prospective analysis plan (see S1 Protocol), we evaluated three hypotheses: (1) penile HIV shedding correlates with wound healing, (2) the frequency and quantity of penile HIV shedding correlate with plasma VL, and (3) ART suppression of HIV plasma VL is associated with decreased penile HIV shedding.

The demographic and clinical characteristics of participants at baseline were tabulated by (1) self-reported not on ART, (2) self-reported ART use with detectable plasma VL, and (3) self-reported ART use with undetectable VL. Differences between groups were estimated using chi-square and Wilcoxon-Mann-Whitney tests for categorical and continuous variables, respectively. The categories for VL and CD4 count were decided using clinically accepted thresholds [27].

The probability of detectable penile HIV shedding at each visit (and 95% CI) for each treatment group was estimated using a modified Poisson regression model with generalized estimating equations (GEE) and a robust variance estimator [28]. This model included main effects for treatment status and weekly visit only. Interaction terms between treatment and visit were not included because no penile shedding was observed at several weekly visits among those who self-reported ART use. While logistic regression is frequently used for binary outcome analyses, modified Poisson regression is the preferred method to estimate relative risk when the outcome is common (>10%) because the odds ratio does not approximate to the relative risk for frequent outcomes [28–30]. However, the results were similar when using either logistic regression or Poisson regression (S1 Fig; S1 Table). We felt that the Poisson estimates were preferable because they are more conservative and more closely approximated the unadjusted risk estimates.

In a separate model, we estimated the prevalence risk ratio (PRR) of detectable lavage HIV-1 relative to baseline among participants not on ART using a modified Poisson regression model with GEE and a robust variance estimator. This secondary analysis included only data from participants not on ART and included only a main effect for visit.

Differences between time to wound healing and time to resumption of sexual intercourse were assessed using the log-rank test for survival data. We also tested for differences in absolute levels of lavage HIV VL (log_10_ copies/milliliter of lavage fluid) among those with detectable lavage HIV using Wilcoxon-Mann-Whitney tests. We compared groups stratified by study visit, ART/VL group, and plasma VL at the time of lavage.

We also analyzed risk factors for a detectable lavage HIV at weekly visits after MC (1–4, 6, and 12 wk post-MC) using modified Poisson regression models and GEE robust variance estimators. We excluded baseline data from these analyses since risk factors for penile HIV shedding after MC may be different from the risk factors before MC. The adjusted analysis included the primary exposure variable, plasma VL, and potential confounding variables. Confounders included those covariates that were associated with plasma VL and penile HIV shedding after MC in univariate analyses at *p<*0.10. In addition to plasma VL, the final adjusted model included baseline CD4 count, wound healing status, treatment with co-trimoxazole, and penile HIV shedding status at baseline. Resumption of sex and ART status variables were not included in our adjusted model because of collinearity with wound healing status and plasma VL, respectively. Religion and condom use and number of sex partners in the last year prior to MC were not associated with penile HIV shedding after MC and were not included the final adjusted model. All analyses were conducted in R version 3.0.1 using the “geepack” package.

### Study Power

Based on preliminary observations, the study was designed to detect whether post-MC HIV wound shedding was associated with plasma VL. Among a proposed population of 131 HIV-infected men, we expected that 116 men would have a detectable plasma VL at the time of MC. The study was designed to be able to detect with 80% power and two-sided alpha = 0.05 whether men with detectable plasma VL were 4.4 times more likely to have HIV wound shedding post-surgery compared to those without detectable HIV plasma VL. In addition, we expected that HIV shedding from MC wounds would decrease with wound healing and would depend on pre-surgical HIV plasma VL. Assuming a standard deviation of log_10_ plasma VL of 0.74 (from preliminary data during the grant submission), the study was designed to detect with 80% power and two-sided alpha = 0.05 a risk ratio of 0.7 with a one-unit increase in log_10_ plasma VL. While the study was initially powered to evaluate 131 HIV-infected men, other outcomes (e.g., safety of MC and wound healing) required a larger sample size and a longer follow-up interval. Thus, we included all consenting HIV-infected men in this study rather than randomly sampling the study population to include 131 HIV-infected men as originally planned.

## Results

### Study Participants

**Fig 1:**
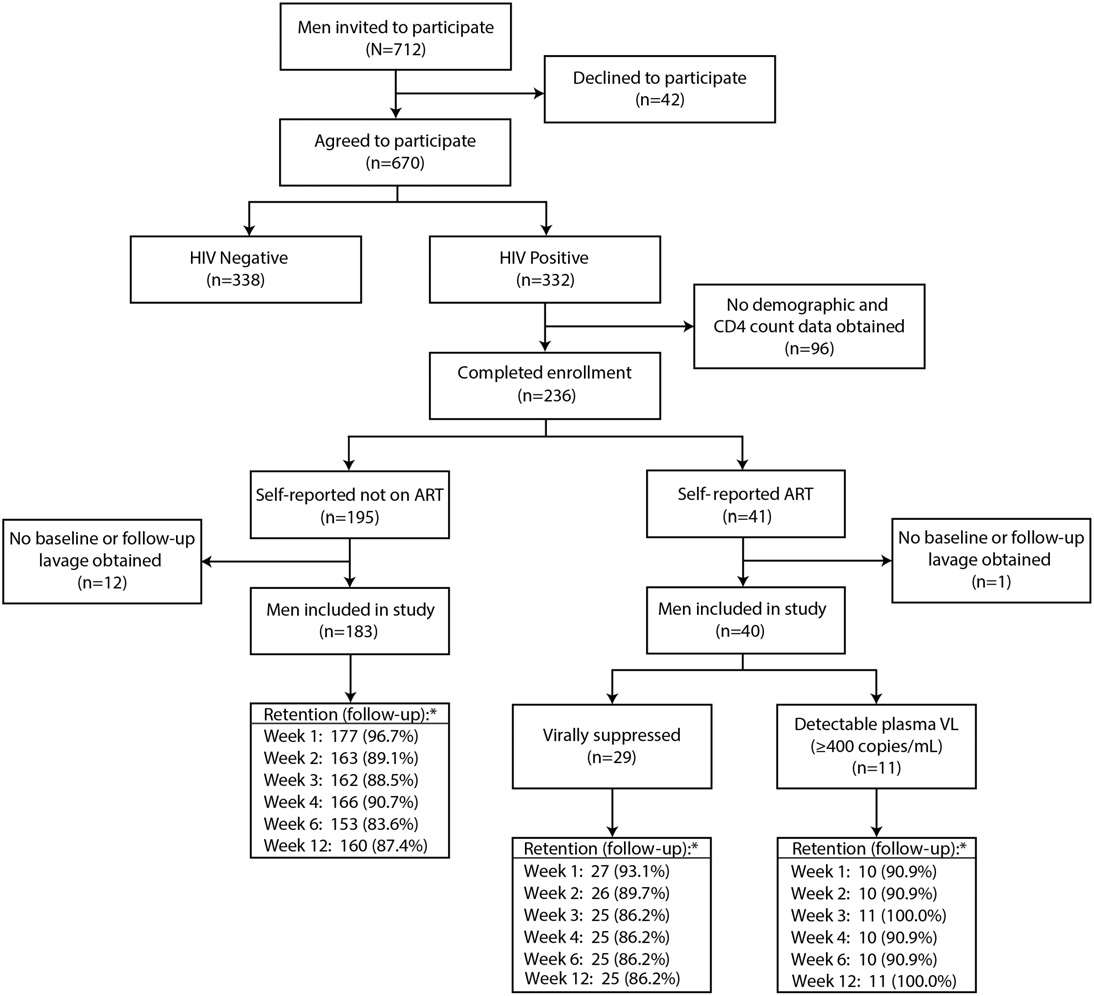
Study flow diagram. The retention (asterisk) indicates the number (percent) of men at each follow-up visit.

There were 712 HIV-negative and HIV-infected men invited to participate. HIV-negative men were enrolled in a parallel study to mask HIV status in the HIV-infected cohort. There were 332 HIV-infected men who agreed to participate in the study (Fig 1). The first 96 HIV-infected men who agreed to participate were excluded since no demographic or CD4 count data were collected at the beginning of the study. Of the 236 men who completed enrollment, 195 (82.6%) were not on ART, and 183 (93.8%) of these men had a baseline and at least one follow-up lavage sample. Among the 41 men (17.4%) who reported ART use, 40 (97.6%) had a baseline and at least one-follow-up lavage sample. Of these 40 men, 29 (72.5%) had undetectable plasma VL and 11 (27.5%) had a detectable plasma VL at enrollment. Thus, there were 223 men included in the analysis.

Penile HIV shedding was evaluated for 96.9% (216/223) of male participants at the surgical visit immediately after MC and at 89.4% (1,196/1,338) of the scheduled weekly visits after MC through week 12. Individuals who missed weekly follow-up visits tended to be younger and Catholic and were less likely to have detectable penile HIV shedding at baseline. Neither self-reported ART status nor baseline CD4 was statistically significantly associated with there being missing outcome data (S2 Table).

The three ART/VL groups of men had similar distributions of age, marital status, and presence of GUD (Table 1). The men who reported ART use had fewer sexual partners, and higher condom and co-trimoxazole use than men not on ART. The median CD4 count was 466 cells/μl (interquartile range [IQR] = 314–655) in men not on ART, 358 cells/μl (IQR = 258–534) in men reporting ART use with an undetectable plasma VL, and 181 cells/μl (IQR = 81–249) for men reporting ART use but with a detectable plasma VL. The median plasma VLs were comparable in the men not on ART and the men on ART with detectable VL. Among men not on ART, 17 (9.3%) had undetectable VL at enrollment.

**Table 1:** Enrollment characteristics.

Characteristic	Total (*n =* 223)	Self-Reported Not on ART (*n =* 183)	Self-Reported on ART		*p*-Value^
			Detectable VL (*n =* 11)	Undetectable VL (*n =* 29)	
**Enrollment age (years)**					0.389
<30	80 (35.9%)	71 (38.8%)	3 (27.3%)	6 (20.7%)	
30–39	102 (45.7%)	79 (43.2%)	6 (54.5%)	17 (58.6%)	
>40	41 (18.4%)	33 (18.0%)	2 (18.2%)	6 (20.7%)	
**Marital Status**					0.529
Not married	62 (27.8)	49 (26.8%)	5 (45.5%)	8 (27.6%)	
Monogamous	145 (65.0%)	119 (65.0%)	6 (54.5%)	20 (69.0%)	
Polygamous	16 (7.2%)	15 (8.2%)	0 (0.0%)	1 (3.4%)	
**Religion**					0.020
Catholic	159 (71.3%)	135 (73.8%)	5 (45.5%)	19 (65.5%)	
Protestant	51 (22.9%)	39 (21.3%)	3 (27.3%)	9 (31.0%)	
Pentecostal or other	13 (5.8%)	9 (4.9%)	3 (27.3%)	1 (3.4%)	
**Number of sexual partners during past year**					0.005
None	14 (6.3%)	8 (4.4%)	1 (9.1%)	5 (17.2%)	
1	78 (35.0%)	58 (31.7%)	7 (63.6%)	13 (44.8%)	
2	68 (30.5%)	57 (31.1%)	3 (27.3%)	8 (27.6%)	
3+	63 (28.3%)	60 (32.8%)	0 (0.0%)	3 (10.3%)	
Condom use during past year*					0.020
None	116 (56.9%)	102 (60.0%)	5 (50%)	9 (37.5%)	
Inconsistent use	63 (30.9%)	53 (31.2%)	3 (30.0%)	7 (29.2%)	
Consistent use	25 (12.3%)	15 (8.8%)	2 (20.0%)	8 (33.3%)	
**GUD during last 30 d**					0.504
No	193 (86.5%)	157 (85.8%)	9 (81.8%)	27 (93.1%)	
Yes	30 (13.5%)	26 (14.2%)	2 (18.2%)	2 (6.9%)	
**Treatment with co-trimoxazole**					<0.001
No	149 (66.8%)	142 (77.6%)	2 (18.2%)	5 (17.2%)	
Yes	74 (33.2%)	41 (22.4%)	9 (81.8%)	24 (82.8%)	
**CD4 count (cells/**μ**l)**					<0.001
<200	28 (12.6%)	17 (9.3%)	6 (54.5%)	5 (17.2%)	
200–500	104 (46.6%)	85 (46.4%)	4 (36.4%)	15 (51.7%)	
>500	91 (40.8%)	81 (44.3%)	1 (9.1%)	9 (31.0%)	
**Median CD4 cell count (IQR) (cells/μl)**	433 (275–625)	466 (314–655)	181 (81–249)	358 (258–534)	<0.001
**Median viral load (IQR) (log copies/ml)**	4.6 (4.2–5.2)	4.6 (4.2–5.2)	4.98 (4.2–5.3)		0.270
**Suppressed viral load (<400 copies/ml)**	46 (20.6%)	17 (9.3%)	0 (0.0%)	29 (100.0%)	

Data are given as number (percent) or median (IQR).

^^^ *p*-Values compare self-reported not on ART, self-reported on ART with detectable VL, and self-reported on ART with undetectable VL.

*Assessed only among sexually active men. Data were also missing for five individuals in the no ART group.

### HIV Shedding from Male Circumcision Wounds

Among all men, penile HIV shedding was detected among 1.9% (5/263) of the weekly visits in men with an undetectable plasma VL and among 14.0% (123/877) of the weekly visits in men with a detectable plasma VL (PRR = 0.13, 95% CI = 0.06–0.31). In multivariate analyses, HIV shedding from MC wounds occurred in more study visits among men with an HIV plasma VL >50,000 copies/ml than among those with an undetectable VL (adjusted PRR [adjPRR] = 10.3, 95% CI = 4.25–24.90, *p<*0.001) (Table 2). HIV shedding was less common in visits from men with healed MC wounds compared to those without fully healed wounds (adjPRR = 0.12, 95% CI = 0.07–0.23, *p<*001). Of the 14 visits at which shedding was detected at or after certified wound healing, 8 (57%) were at the visit when wound healing was certified. HIV shedding was also less common in visits from men who reported ART use and had an undetectable plasma VL compared to men not on ART (PRR = 0.15, 95% CI = 0.05–0.43, *p =* 0.001), and this difference remained statistically significant even after adjusting for treatment with co-trimoxazole, healed wounds, HIV shedding prior to MC, and baseline CD4 count (adjPRR = 0.13, 95% CI = 0.04–0.45, *p =* 0.001).

**Table 2:** Associations with the detection of penile HIV shedding.

Characteristic	Number of Visits with HIV Shedding/Total Visits	Percent of Visits with HIV Shedding	PRR (95% CI)	*p*-Value	adjPRR* (95% CI)	*p*-Value
**Age (years)**						
<30	46/415	11.1%	1.00 (referent)	—	—	—
30–39	63/556	11.3%	1.02 (0.63–1.65)	0.942	—	—
>40	24/225	10.7%	0.96 (0.53–1.73)	0.890	—	—
**GUD**						
No	115/1,040	11.1%	1.00 (referent)	—	—	—
Yes	18/156	11.5%	1.07 (0.63–1.82)	0.812	—	—
**Treatment with co-trimoxazole**						
No	102/795	12.8%	1.00 (referent)	—	1.00 (referent)	
Yes	31/401	7.7%	0.60 (0.37–0.97)	0.035	0.86 (0.55–1.33)	0.497
**Certified healed wound** †						
No	118/609	19.4%	1.00 (referent)	—	1.00 (referent)	
Yes	14/569	2.5%	0.14 (0.06–0.33)	<0.001	0.12 (0.07–0.23)	<0.001
**Resumed sex in week prior** †						
No	124/856	14.5%	1.00 (referent)	—	—	—
Yes	7/309	2.3%	0.12 (0.04–0.46)	<0.001	—	—
**HIV shedding prior to MC**						
No	108/1,094	9.9%	1.00 (referent)	—	1.00 (referent)	
Yes	25/102	24.5%	2.49 (1.49–4.18)	<0.001	1.62 (0.98–2.65)	0.057
**Baseline CD4 count (cells/μl)**						
>500	45/487	9.2%	1.00 (referent)	—	1.00 (referent)	—
200–500	64/554	11.6%	1.25 (0.76–2.03)	0.361	1.01 (0.64–1.60)	0.965
<200	24/155	15.5%	1.62 (0.92–2.85)	0.092	1.08 (0.63–1.59)	0.773
**Baseline plasma VL (copies/ml)** †						
<400	5/263	1.9%	1.00 (referent)	—	1.00 (referent)	—
400–9,999	13/216	6.0%	3.61 (1.40–9.28)	0.008	3.36 (1.25–9.04)	0.016
10,000–49,000	26/261	10.0%	5.36 (2.14–13.40)	0.003	5.47 (2.19–13.60)	<0.001
>50,000	84/400	21.0%	11.10 (4.67–26.50)	<0.001	10.30 (4.25–24.90)	<0.001
**Self-reported ART and VL**						
Not on ART	126/981	12.8%	1.00 (referent)	—	—	—
On ART with detectable VL	4/62	6.5%	0.49 (0.17–1.41)	0.185	—	—
On ART with suppressed VL	3/153	2.0%	0.15 (0.05–0.43)	0.001	—	—

*Adjusted analysis included the following variables: treatment with co-trimoxazole, certified healed wound, HIV shedding prior to MC, baseline CD4 count, and plasma VL. Resumption of sexual intercourse and self-reported ART status were not included in the adjusted analysis because of co-linearity with certified wound healing and plasma VL, respectively.

^†^There were 18 visits (1.4%) with missing wound-healing information, 31 visits with missing information on resumption of sexual intercourse, and 56 visits (4.7%) with missing plasma VL data. The adjusted analysis was a complete case analysis and included 98.5% of observed visits after MC (1,178/1,196).

### HIV Shedding from Male Circumcision Wounds Stratified by ART Status

**Fig 2:**
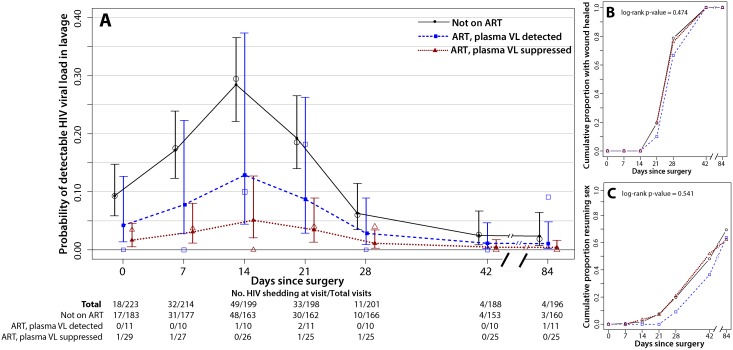
Penile HIV shedding, wound healing, and resumption of sex stratified by plasma viral load and ART status. The day 0 visit is prior to surgery. (A) HIV shedding from MC wounds increased at 7, 14, and 21 d after MC. The open symbols are the empirical data, and the filled symbols and line are the fit estimates. The error bars represent the 95% CIs of the fitted values. (B) There was no difference in certified wound healing between the three ART/VL groups. (C) There was no difference in the proportion of men resuming sex between the three ART/VL groups.

Among men not on ART, penile HIV shedding was detected at 12.8% (126/981) of the weekly visits after MC and in 39.3% (72/183) of the men (Fig 2A). Prior to MC, HIV shedding was detected in 9.3% (17/183) of men not on ART, and during surgery HIV shedding was detected in 57.6% (102/177) of these men. Relative to baseline, the probability of detectable HIV shedding was significantly increased after MC at 1 wk (17.5% [31/177], PRR = 1.87, 95% CI = 1.12–3.14, *p =* 0.012), 2 wk (29.4% [48/163], PRR = 3.16, 95% CI = 1.94–5.13, *p<*0.001), and 3 wk (18.5% [30/162], PRR = 1.98, 95% CI = 1.19–3.28, *p =* 0.008). However, detectable HIV shedding was significantly lower than at baseline by 6 wk after MC (2.6% [4/153], PRR = 0.27, 95% CI = 0.09–0.83, *p =* 0.023). Twelve weeks after MC the proportion of men not on ART with detectable penile HIV shedding was only 1.9% (3/160, PRR = 0.19, 95% CI = 0.06–0.64, *p =* 0.008).

Men not on ART at baseline may have initiated treatment during the study. However, among the 165 men who self-reported not being on ART and who had a detectable plasma VL at enrollment, only nine (5.4%) had an undetectable plasma VL at their final follow-up visit. In a sensitivity analysis restricted to men who self-reported not being on ART and who had a detectable plasma VL both at enrollment and at the final follow-up visit, the probability of penile HIV shedding was still significantly reduced at 6 wk (PRR = 0.21, 95% CI = 0.06–0.75, *p =* 0.017) and 12 wk (PRR = 0.21, 95% CI = 0.06–0.70, *p =* 0.011).

Among the 11 men who reported ART use but had a detectable plasma VL at enrollment, three men (27.3%) had detectable penile HIV shedding, detected at four of the total of 62 weekly visits after MC (6.5%) (Fig 2A). Prior to MC, no HIV shedding was detected among these men, but during surgery, HIV shedding was detected among 45.5% (5/11) of these men. The probability of detectable shedding at any weekly visit after MC was not significantly lower than in men not on ART (PRR = 0.49, 95% CI = 0.17–1.41, *p =* 0.185) (Table 2). Two of the 11 men who reported ART use but had a detectable plasma VL at enrollment then had undetectable plasma VL by 6 wk. Both of these men also had undetectable plasma VL at 12 wk; however, one man had detectable penile HIV shedding at his final study visit.

Among the 29 men who reported ART use and had an undetectable plasma VL at baseline, there were three men (10.3%) with HIV shedding, detected at three of the total of 153 weekly visits after MC (2.0%) (Fig 2A). Prior to MC, HIV shedding was detected among 3.4% (1/29) of these men, and during surgery, HIV shedding was detected among 28.6% (8/28) of these men. The probability of detectable HIV shedding was significantly lower after MC for men reporting ART use who had an undetectable VL at baseline than for men not on ART (PRR = 0.15, 95% CI = 0.05–0.43, *p =* 0.001) (Table 2).

### Wound Healing and Resumption of Sex

Time to wound healing and time to resumption of sexual intercourse did not vary between the three ART/VL groups (*p =* 0.474 and *p =* 0.541, respectively) (Fig 2B and 2C). All of the men in the study were fully healed by 6 wk post-MC (Fig 2B); 48% (*n =* 107) of the men in the study resumed sexual intercourse prior to 6 wk post-MC, and this did not vary by ART/VL group (Fig 2C).

### HIV Viral Load from Male Circumcision Wounds

**Fig 3:**
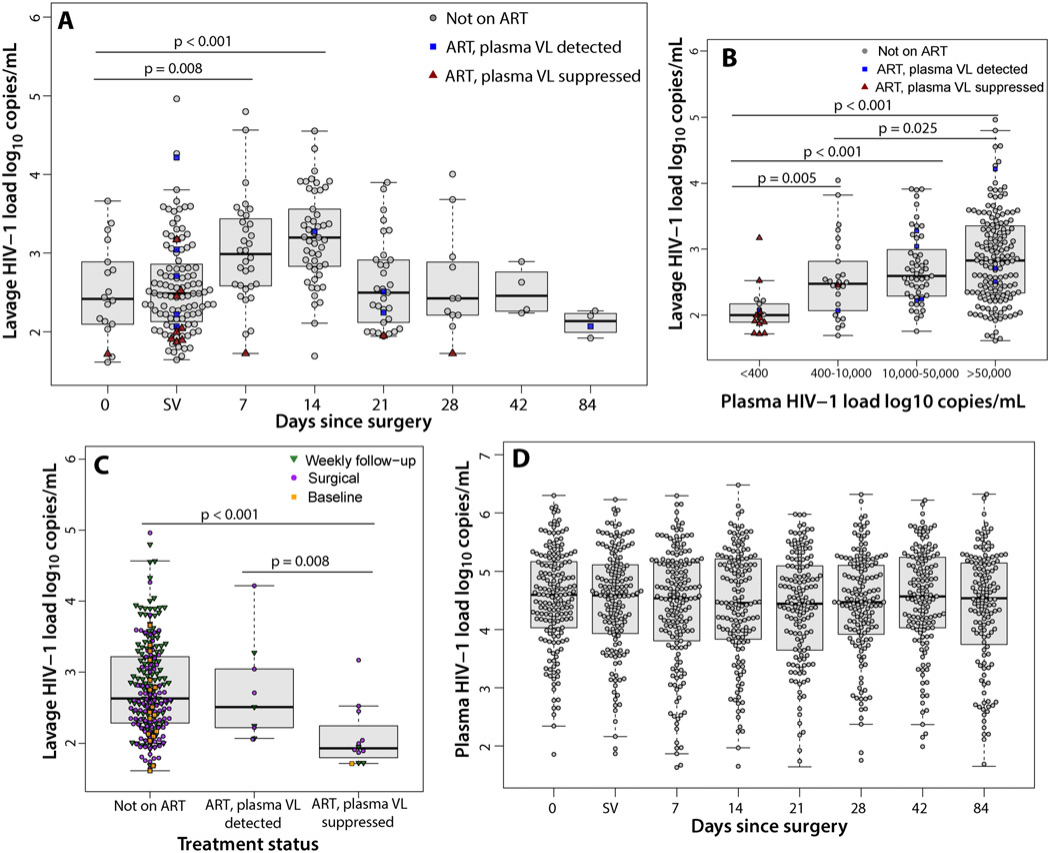
Penile shedding HIV viral load stratified by ART status and plasma viral load. (A) The median log_10_ penile shedding VL increased at 7 and 14 d after surgery. Day 0 represents the visit prior to MC. SV represents the surgical visit. (B) The median log_10_ penile shedding VL is associated with increasing plasma VL. (C) The median log_10_ penile shedding VL is decreased among men on ART with an undetectable plasma VL. (D) The median log_10_ plasma VL does not increase after MC.

Among all men with detectable penile HIV shedding, the median log_10_ HIV shedding was significantly higher at 1 wk (2.99, IQR = 2.59–3.02, *p* = 0.008) and 2 wk (3.20, IQR = 2.83–3.56, *p* = 0.008) compared to prior to surgery (2.42, IQR = 2.11–2.86) or during surgery immediately following foreskin removal (2.49, IQR = 2.13–2.58) (Fig 3A). The median log_10_ penile HIV shedding increased significantly with higher plasma VL (Figs 3B and S2).

The median log_10_ penile HIV shedding was significantly lower among men on ART who had an undetectable plasma VL (1.93, IQR = 1.83–2.14) compared to men not on ART (2.63, IQR = 2.28–3.22, *p<*0.001) or men with reported ART use but who had a detectable plasma VL (2.51, IQR = 2.22–3.04, *p =* 0.008) (Fig 3C). There were no significant increases in plasma VL after MC (Fig 3D). Among men who reported ART use at baseline and had an undetectable plasma VL, only 3.4% (1/29) had detectable plasma VL after MC.

## Discussion

HIV shedding from MC wounds was detected among 39% of HIV-infected men who reported not being on ART during the wound healing phase, but was detected among a significantly smaller number of individuals who reported ART use and had an undetectable plasma VL. HIV shedding was most common among men without complete wound healing. To our knowledge, this is the first study to suggest that ART decreases both the frequency of detected HIV shedding and the quantity of virus shed from MC wounds.

HIV shedding at 6 and 12 wk post-MC, when all MC wounds had healed, was significantly lower than prior to MC, as reported in one previous study [25]. These data support observational studies and mathematical models suggesting that MC performed prior to men’s sexual debut (i.e., before they become sexually active) may have long-term benefits for decreasing HIV transmission to women [18,19,23,31]. The decreased shedding 6 wk after MC may be due to the removal of the foreskin and formation of an intact scar.

While there was no change in plasma VL post-MC, penile HIV shedding VL increased at 1 and 2 wk after MC, even compared to samples obtained during surgery that were clearly contaminated with blood. This increase may be due to inflammation and recruitment of HIV target cells during wound healing [32,33].

This study has limitations. Only 73% of men self-reporting ART use had an undetectable plasma VL, similar to rates seen elsewhere in Africa and in developed countries [34,35]. Since the large majority of men received ART outside of our care program, we were unable to confirm actual receipt of ART. In addition, men self-reported whether they were currently taking ART; it is unknown whether men had previously taken ART or recently stopped ART. However, >90% of men who did not report ART use had a detectable plasma VL. The standard ART regimen in Uganda is efavirenz/lamivudine/tenofovir and has been reported to reduce plasma VL to <400 copies/ml in 55% of individuals within 2 wk of initiation, and in 65% of individuals by 4 wk [36,37]. However, only nine men not on ART (5.4%) in this study had an undetectable plasma VL after having a detectable plasma VL at enrollment. In a sensitivity analysis that evaluated men not on ART who had a detectable plasma VL both at enrollment and at the final follow-up visit, the probability of penile HIV shedding remained significantly lower at 6 and 12 wk compared to prior to MC. In addition, the proportion of men with an undetectable plasma VL does not affect the finding that men who were virally suppressed by ART had significantly fewer instances of HIV shedding and shed at a lower VL. Due to the observational nature of this study, the findings are associations and do not necessarily imply causality. Another limitation of this observational study is that there were significant differences in covariates at enrollment, which raises the possibility of residual uncontrolled confounding. The rates of HIV shedding from MC wounds presented here are lower than in one previous report [25]. This could be due to differences in sample collection, assay sensitivity, surgical procedure, or plasma VL.

Ideally, MC programs in sub-Saharan Africa should target HIV-negative men to prevent HIV acquisition, although it is inevitable that some HIV-infected men will request the procedure. WHO recommends that although MC should not be promoted for HIV-infected men, they should not be denied MC unless they have contraindications for surgery [21]. Programs designed to provide MC prior to sexual debut (such as in early adolescence or as a neonate, at which age only a very small proportion of African males are HIV-infected or sexually active) would avoid potential problems of HIV transmission during wound healing [38,39] and are cost-effective [40]. However, in reality, programs offering MC to adult men will remain an important HIV-prevention priority for the foreseeable future in order decrease HIV incidence as rapidly as possible. Approximately 6% of all men requesting MC are HIV-infected [23]. Because MC is becoming more normative in regions where it is promoted, it is likely that more HIV-infected men will request the procedure. A mathematical model evaluating MC in a general population of HIV-negative and HIV-infected men living in sub-Saharan Africa, with a proportion having sexual intercourse during the MC wound healing phase, predicted an overall 0.06% increase in HIV transmission to female partners [23]. With the WHO goal to circumcise 28.7 million men [41], this could lead to 17,000 new infections in female partners.

We do not know the impact of penile HIV shedding from MC wounds on actual transmission to female partners, but higher plasma VL increases transmission risk [27], as does HIV shedding from genital ulcers [42,43]. Thus, it is plausible, and supported by the trial of MC in HIV-infected men [17], that viral shedding from a post-MC wound increases risk of transmission. A large randomized trial enrolling both men and female partners with long-term follow-up would be needed to confirm these results, but this type of trial may be logistically unfeasible. However, it is important to ensure that HIV transmission to female partners in the immediate post-surgical period is avoided. The findings from this study reinforce the need for MC programs to provide voluntary HIV counseling and testing prior to MC, promote sexual abstinence during wound healing and condom use thereafter, offer free condoms, and encourage counseling for HIV-infected men and their sexual partners on the risk of HIV transmission if sexual intercourse is resumed prior to complete wound healing.

While additional studies would need to be performed, we believe that consideration should be given to initiating ART prior to MC for HIV-infected men and encouraging adherence to suppress the plasma VL. This strategy would be similar to strategies employed for prevention of mother-to-child transmission. Initiating ART at time of MC or prior to MC could be difficult for MC programs, particularly those employing mobile MC camps. However, with recent WHO guidelines increasing the CD4 count treatment initiation threshold to ≤500 cells/μl [44], the majority of HIV-infected men seeking MC would already qualify for continued lifelong ART [45]. Men underutilize ART in sub-Saharan Africa [46], so incorporating ART treatment referral into MC programs may increase utilization, improve the health of these men, protect their female partners, and help integrate the health care delivery systems in sub-Saharan Africa. However, further research is needed to investigate the acceptability of, feasibility of, and time required for men initiating ART prior to MC to reduce HIV shedding from MC wounds.

## Supporting Information

S1 Checklist STROBE checklist statement. (DOCX)

S1 Fig Probability of detectable penile HIV shedding at baseline and at each weekly follow-up visit using both Poisson and logistic regression models with generalized estimating equations and robust variance estimators. The estimated probabilities of penile HIV shedding from the Poisson model at baseline and at each weekly visit were virtually identical to those obtained from the logistic model. Furthermore, no estimated probability (or upper or lower confidence bound) exceeded one in the Poisson analysis.
(TIF)

S2 Fig Plasma viral load compared to lavage viral load among men who had detectable virus in both the plasma and lavage fluid. (TIFF)

S1 Protocol Prospective analysis plan. (DOCX)

S1 Table Comparison of adjusted relative risks (Table 2) between Poisson and logistic regression models with generalized estimating equations and robust variance estimators. The odds ratios and 95% CIs estimated from the logistic regression models were more extreme than the risk ratios estimated from the Poisson models, as would be expected for common outcomes such as in this study.
(DOCX)

S2 Table Risk factors for a missed weekly follow-up visit after male circumcision. (DOCX)

S1 Text Rakai Health Sciences Program Investigators. (DOCX)

## Abbreviations

adjPRR: adjusted prevalence risk ratio
ART: antiretroviral therapy
GEE: generalized estimating equations
GUD: genital ulcer disease
HPV: human papillomavirus
IQR: interquartile range
MC: (voluntary medical) male circumcision
PRR: prevalence risk ratio
RT-PCR: reverse transcription PCR
VL: viral load
WHO: World Health Organization
