# Supplementary material for: HIV Shedding from Male Circumcision Wounds in HIV-Infected Men: A Prospective Cohort Study
Source: PLoS Med. 2015 Apr 28;12(4):e1001820. doi: 10.1371/journal.pmed.1001820 (PMC4412625; doi:10.1371/journal.pmed.1001820)
Supplement: S1 Checklist — (DOCX) [file pmed.1001820.s001.docx]

STROBE Statement—checklist of items that should be included in reports of observational studies

|  | Item No. | Recommendation | Page  No. | Relevant text from manuscript |
| --- | --- | --- | --- | --- |
| **Title and abstract** | 1 | (*a*) Indicate the study’s design with a commonly used term in the title or the abstract | 1 | We conducted a prospective observational study in Rakai, Uganda to assess HIV shedding post-male circumcision (MC). |
|  |  | (*b*) Provide in the abstract an informative and balanced summary of what was done and what was found | 2-3 | Penile HIV shedding is significantly reduced after healing of MC wounds in ART-naïve men. Lower plasma VL is associated with decreased frequency and quantity of HIV shedding from MC wounds. Starting ART prior to MC should be considered to reduce male-to-female HIV transmission risk. |
| Introduction | | | |  |
| Background/rationale | 2 | Explain the scientific background and rationale for the investigation being reported | 4-5 | To our knowledge, the association of HIV shedding from MC wounds and plasma viral load (VL), and the potential impact of antiretroviral therapy (ART) on HIV shedding have not been assessed. |
| Objectives | 3 | State specific objectives, including any prespecified hypotheses | 5 | Here we report the prevalence and amount of HIV shedding following MC in HIV-infected men with and without ART to assess the a priori hypotheses that penile HIV shedding from MC wounds increases immediately after surgery and the quantity of shedding is dependent on plasma VL. |
| Methods | | | |  |
| Study design | 4 | Present key elements of study design early in the paper | 6-8 | A prospective observational study was conducted between June 2009 and April 2012 in Rakai, Uganda to assess HIV shedding from MC wounds. |
| Setting | 5 | Describe the setting, locations, and relevant dates, including periods of recruitment, exposure, follow-up, and data collection | 6 | A prospective observational study was conducted between June 2009 and April 2012 in Rakai, Uganda to assess HIV shedding from MC wounds. |
| Participants | 6 | (*a*) *Cohort study*—Give the eligibility criteria, and the sources and methods of selection of participants. Describe methods of follow-up  *Case-control study*—Give the eligibility criteria, and the sources and methods of case ascertainment and control selection. Give the rationale for the choice of cases and controls  *Cross-sectional study*—Give the eligibility criteria, and the sources and methods of selection of participants | 6-8 | Uncircumcised HIV-infected men aged ≥15 who requested free MC services and had no contraindication to surgery were invited to participate. All participants provided written informed consent prior to enrollment. |
|  |  | (*b*) *Cohort study*—For matched studies, give matching criteria and number of exposed and unexposed  *Case-control study*—For matched studies, give matching criteria and the number of controls per case | N/A |  |
| Variables | 7 | Clearly define all outcomes, exposures, predictors, potential confounders, and effect modifiers. Give diagnostic criteria, if applicable | 9-10 | The adjusted analysis included the primary exposure variable, plasma VL, and potential confounding variables. Confounders included those covariates that were associated with treatment status and penile HIV shedding after MC in univariate analyses at p<0.10. |
| Data sources/ measurement | 8* | For each variable of interest, give sources of data and details of methods of assessment (measurement). Describe comparability of assessment methods if there is more than one group | *7* | Information on sexual behaviors and blood for plasma VL testing were obtained at each follow-up visit. Would healing was assessed via direct observation starting at the three–week post-operative visit by clinical officers. Certified complete wound healing was defined as an intact scar with no scab, sutures or stitch sinus.  Samples to assess HIV viral shedding at the coronal sulcus prior to MC and at the MC surgical site were also collected. |
| Bias | 9 | Describe any efforts to address potential sources of bias | 8-10 | An additional sensitivity analysis was added to assess possible bias of initiating ART after study enrolment. |
| Study size | 10 | Explain how the study size was arrived at | 11 | Based on a preliminary observation, the study was designed to detect whether post-MC HIV wound shedding was associated with plasma VL. Among a proposed population of 131 HIV-infected men, we expected 116 men would have a detectable plasma VL at time of MC. With 80% power and two-sided alpha=0.05, the study was designed to be able to detect whether men with detectable plasma VL were 4.4 times more likely to have HIV wound shedding post-surgery compared to those without detectable HIV plasma VL. In addition, we expected that HIV shedding from MC wounds decreases with wound healing and depends on pre-surgical HIV plasma VL. Assuming a standard deviation of log_10_ plasma VL is 0.74 (from preliminary data during the grant submission), with 80% power and two-sided alpha=0.05, the study had the power to detect a risk ratio of 0.7 with one unit increase in log_10_ plasma VL. While the study was initially powered to evaluate 131 HIV-infected men, other outcomes (e.g., safety of MC and wound healing) required a larger sample size and a longer follow-up interval. Thus, we included all consenting HIV-infected men in this study rather than randomly sampling the study population to include 131 HIV-infected men as originally planned. |

Continued on next page

| Quantitative variables | | 11 | | Explain how quantitative variables were handled in the analyses. If applicable, describe which groupings were chosen and why | 8-9 | | Demographic and clinical characteristics of participants at baseline were tabulated by: (1) self-reported ART-naïve, (2) self-reported ART with detectable plasma VL, and (3) self-reported ART with undetectable VL. Differences between groups were estimated using Chi-square and Wilcoxon-Mann Whitney tests for categorical and continuous variables, respectively. |
| --- | --- | --- | --- | --- | --- | --- | --- |
| Statistical methods | | 12 | | (*a*) Describe all statistical methods, including those used to control for confounding | 8-10 | | The probability of detectable penile HIV shedding at each visit (and 95% CI) for each treatment group was estimated using a modified Poisson regression model with generalized estimating equations (GEE) and a robust variance estimator. In a separate model, we estimated the prevalence risk ratio (PRR) of detectable lavage HIV-1 relative to baseline among non-ART users also using a modified Poisson regression model with generalized estimating equations (GEE) robust variance estimator. Differences between time to wound healing and resumption of sexual intercourse were assessed using the log-rank test for survival data. We also tested for differences in absolute levels of lavage HIV VL (log_10_ copies/mL of lavage fluid) among those with detectable lavage HIV using Wilcoxon-Mann-Whitney tests. All analyses were conducted in R version 3.0.1 using the “geepack” package. |
|  |  |  |  | (*b*) Describe any methods used to examine subgroups and interactions | 8-10 | | See above |
|  |  |  |  | (*c*) Explain how missing data were addressed | 12 | | Individuals who missed weekly follow-up visits tended to be younger and Catholic and were less likely to have detectable penile HIV shedding at baseline. Neither self-reported ART status nor baseline CD4 were statistically significantly associated with missing outcome data (S2 Table). |
|  |  |  |  | (*d*) *Cohort study*—If applicable, explain how loss to follow-up was addressed  *Case-control study*—If applicable, explain how matching of cases and controls was addressed  *Cross-sectional study*—If applicable, describe analytical methods taking account of sampling strategy | 8-10 | | See above |
|  |  |  |  | (*e*) Describe any sensitivity analyses |  | | N/A |
| Results | | | | | | | |
| Participants | | 13* | | (a) Report numbers of individuals at each stage of study—eg numbers potentially eligible, examined for eligibility, confirmed eligible, included in the study, completing follow-up, and analysed | 12 | | There were 712 HIV-negative and HIV-infected men invited to participate. HIV-negative men were enrolled in a parallel study to mask HIV status. There were 332 HIV-infected men who agreed to participate in the study (Fig. 1). The first 96 HIV-infected men who agreed to participate were excluded since no demographic or CD4 count data were collected at the beginning of the study . Of the 236 men who completed enrollment, 195 (82.6%) were ART-naïve and 183 (93.8%) of these men had a baseline and at least one follow-up sample. Among the 41 men (17.4%) who reported ART use, 40 (97.6%) had a baseline and at least one-follow-up sample. Of these 40 men, 29 (72.5%) had undetectable plasma VL and 11 (27.5%) had a detectable plasma VL at enrollment. Thus, there were 223 men included in the analysis. |
|  |  |  |  | (b) Give reasons for non-participation at each stage |  | | See above |
|  |  |  |  | (c) Consider use of a flow diagram |  | | Figure 1 |
| Descriptive data | | 14* | | (a) Give characteristics of study participants (eg demographic, clinical, social) and information on exposures and potential confounders | 12-13 | | The three groups of men had similar categories of age, marital status and presence of GUD (Table 1). The men who reported ART use had fewer sexual partners, and higher condom and co-trimoxazole use than the ART-naive. The median CD4 count was 466 cells/uL (IQR 314-655) in ART-naive, 358 cells/uL(IQR 258-534) in men reporting ART use with an undetectable plasma VL, and 181 cells/uL (IQR 81-249) for men reporting ART use but with a detectable plasma VL. The median plasma VLs were comparable in the ART-naïve men and men on ART with detectable VL. Among ART-naïve men, 17 (9.3%) had undetectable VL at enrollment. |
|  |  |  |  | (b) Indicate number of participants with missing data for each variable of interest |  | | S2 Table |
|  |  |  |  | (c) *Cohort study*—Summarise follow-up time (eg, average and total amount) | 12 | | Figure 1 |
| Outcome data | | 15* | | *Cohort study*—Report numbers of outcome events or summary measures over time | *11-13* | | Among all men, penile HIV shedding was detected among 1.9% (5/263) of the weekly visits in men with an undetectable plasma VL and among 14.0% (123/877) of the weekly visits in men with a detectable plasma VL (PRR=0.13, 95%CI=0.06-0.31). |
|  |  |  |  | *Case-control study—*Report numbers in each exposure category, or summary measures of exposure |  | | *N/A* |
|  |  |  |  | *Cross-sectional study—*Report numbers of outcome events or summary measures |  | | *N/A* |
| Main results | | 16 | | (*a*) Give unadjusted estimates and, if applicable, confounder-adjusted estimates and their precision (eg, 95% confidence interval). Make clear which confounders were adjusted for and why they were included | 16-17 | | Table 2 |
|  |  |  |  | (*b*) Report category boundaries when continuous variables were categorized |  | | N/A |
|  |  |  |  | (*c*) If relevant, consider translating estimates of relative risk into absolute risk for a meaningful time period |  | | N/A |
| Other analyses | 17 | | Report other analyses done—eg analyses of subgroups and interactions, and sensitivity analyses | | 19-20 | Among the 11 men who reported ART use but had a detectable plasma VL at enrollment, three men (27.3%) had detectable penile HIV shedding at 4 of the 62 weekly visits after MC (6.5%) (Fig. 2A). | |
| Discussion | | | | | | | |
| Key results | 18 | | Summarise key results with reference to study objectives | | 10 | HIV shedding from MC wounds was detected among 39% of ART-naïve HIV-infected men during the wound healing phase, but significantly decreased among individuals with an undetectable plasma VL and reported ART use. HIV shedding was most common among men without complete wound healing. | |
| Limitations | 19 | | Discuss limitations of the study, taking into account sources of potential bias or imprecision. Discuss both direction and magnitude of any potential bias | | 22-23 | This study has limitations. Only 73% of men self-reporting ART-use had an undetectable plasma VL, similar to rates seen in Africa and developed countries. Since the large majority of men received ART outside of our care program, we were unable to confirm actual receipt of ART. The standard ART regimen in Uganda is EFV/3TC/TDF and has been reported to reduce plasma VL <400 copies/mL in 55% of individuals within two weeks of initiation, and for 65% of individuals by week four. However, only nine ART-naïve men (5.4%) in this study had an undetectable plasma VL after having a detectable plasma VL at enrollment. In a sensitivity analysis which evaluated ART-naïve men with detectable plasma VL both at enrollment and the final follow-up visit, penile HIV shedding remained significantly lower at weeks 6 and 12 compared to prior to MC. In addition, the rates of men with an undetectable plasma VL do not affect the finding that men who were virally suppressed by ART had significantly fewer instances of HIV shedding and shed at a lower VL. Due to the observational nature of this study, the findings are associations and do not necessarily imply causality. Another limitation of this observational study is that there were significant differences in covariates at enrollment, which raise the possibility of residual uncontrolled confounding. | |
| Interpretation | 20 | | Give a cautious overall interpretation of results considering objectives, limitations, multiplicity of analyses, results from similar studies, and other relevant evidence | | 22-25 | HIV shedding six and twelve weeks post-MC when all MC wounds had healed was significantly lower than prior to MC, as reported in one previous study. | |
| Generalisability | 21 | | Discuss the generalisability (external validity) of the study results | | 22-25 | To our knowledge, this is the first study to suggest that ART decreases both the number of HIV shedding events and the quantity of virus shed from MC wounds. | |
| Other information | | |  | | | | |
| Funding | 22 | | Give the source of funding and the role of the funders for the present study and, if applicable, for the original study on which the present article is based | |  | This study was supported by the Bill and Melinda Gates Foundation (22006.03, sample collection), the Doris Duke Charitable Foundation (#2011036, laboratory work), the Division of Intramural Research, National Institute of Allergy and Infectious Diseases and the Fogarty International Center, 1D43TWOO9578-01. A.A.R.T. was supported by the NIH 1K23AI093152-01A1 and the Doris Duke Charitable Foundation Clinician Scientist Development Award. M.K.G was supported by NIH T32AI102 and the Doris Duke Charitable Foundation Clinician Scientist Development Award. The funders had no role in study design, data collection and analysis, decision to publish, or preparation of the manuscript. | |

*Give information separately for cases and controls in case-control studies and, if applicable, for exposed and unexposed groups in cohort and cross-sectional studies.

**Note:** An Explanation and Elaboration article discusses each checklist item and gives methodological background and published examples of transparent reporting. The STROBE checklist is best used in conjunction with this article (freely available on the Web sites of PLoS Medicine at http://www.plosmedicine.org/, Annals of Internal Medicine at http://www.annals.org/, and Epidemiology at http://www.epidem.com/). Information on the STROBE Initiative is available at www.strobe-statement.org.
