# Supplementary figures and images for: HIV Shedding from Male Circumcision Wounds in HIV-Infected Men: A Prospective Cohort Study
Source: PLoS Med. 2015 Apr 28;12(4):e1001820. doi: 10.1371/journal.pmed.1001820 (PMC4412625; doi:10.1371/journal.pmed.1001820)

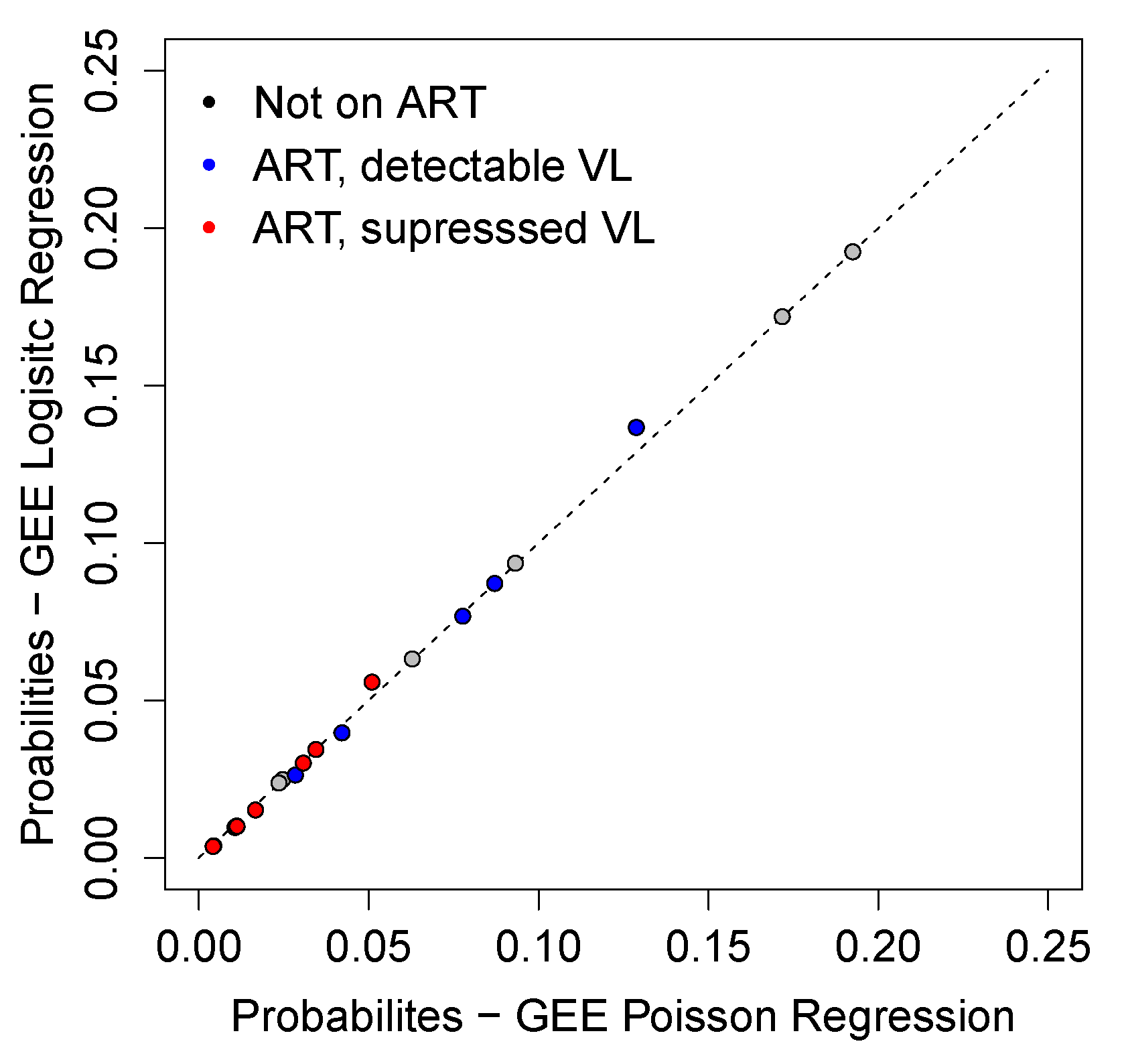

Supplement: S1 Fig — The estimated probabilities of penile HIV shedding from the Poisson model at baseline and at each weekly visit were virtually identical to those obtained from the logistic model. Furthermore, no estimated probability (or upper or lower confidence bound) exceeded one in the Poisson analysis. (TIF) [file pmed.1001820.s002.tif]

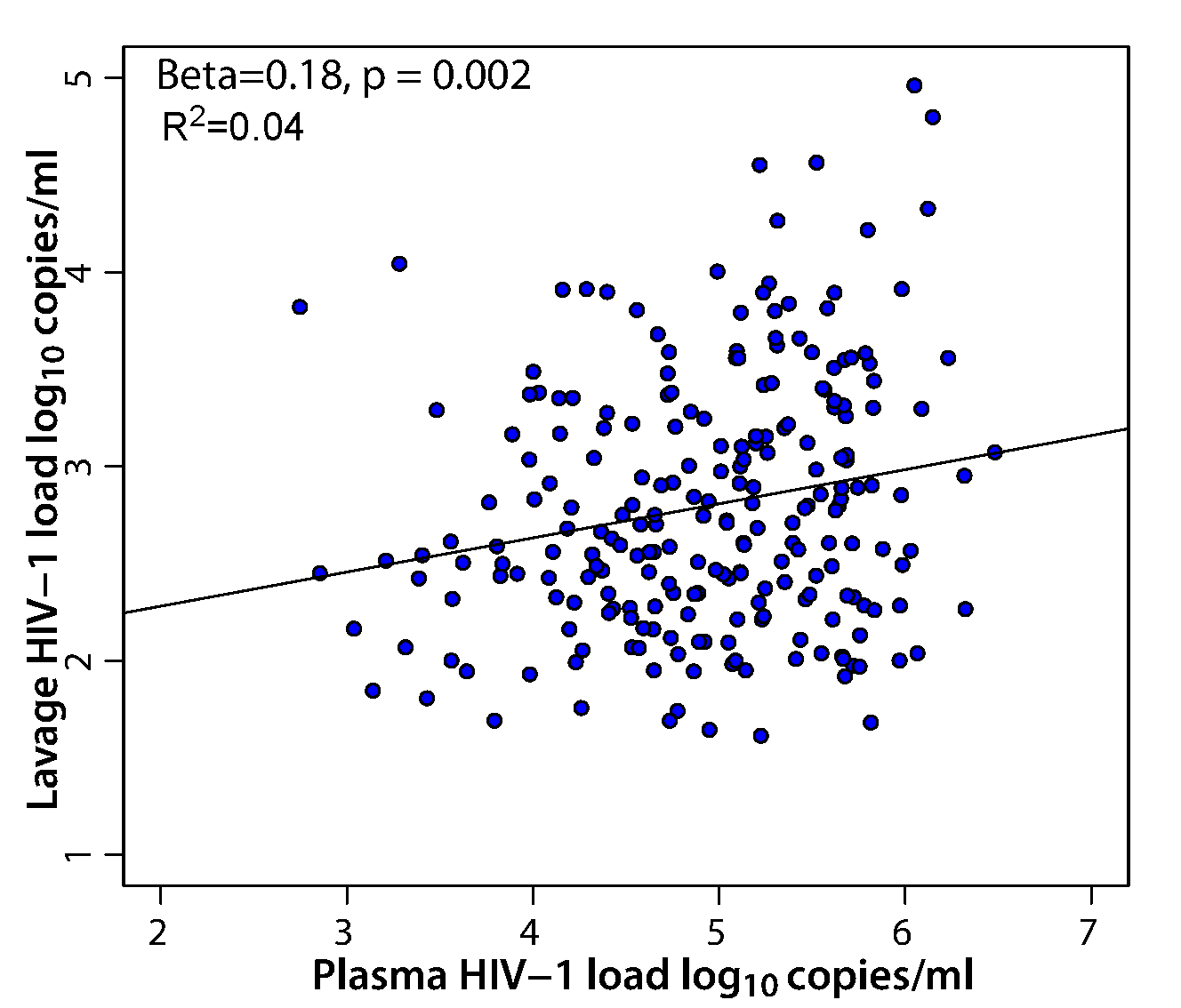

Supplement: S2 Fig — (TIFF) [file pmed.1001820.s003.tiff]
