# Supplementary material for: HIV Shedding from Male Circumcision Wounds in HIV-Infected Men: A Prospective Cohort Study
Source: PLoS Med. 2015 Apr 28;12(4):e1001820. doi: 10.1371/journal.pmed.1001820 (PMC4412625; doi:10.1371/journal.pmed.1001820)
Supplement: S1 Protocol — (DOCX) [file pmed.1001820.s004.docx]

**Prospective Analysis Plan**

**Doris Duke Charitable Foundation Grant: HIV and HSV-2 shedding and transmission in recently circumcised men**

**Funded July 2011**

**Research Plan**

**A. Hypothesis and Specific Aim**

**Hypothesis:** Penile HIV viral wound shedding increase immediately after MC leading to temporarily higher rates of HIV transmission to female partners.

**Aim 1. Aim 1a:** Among 131 HIV-positive men, assess whether HIV shedding occurs in the preputial cavity of uncircumcised HIV-positive men and in healing MC wounds, and to determine whether HIV shedding decreases with wound healing and post-healing scar formation/keratinization. **Aim 1b:** Assess whether penile HIV shedding is associated with HSV-2 serostatus, CD4 counts, HIV viral load, subtype, viral suppressive therapy and time to clinically certified wound healing.

**1a. Study design population and setting.** HIV shedding will be examined for the 131 HIV-positive men with previously collected swabs and wound lavage samples for eight weeks post MC surgery (see preliminary results section D.3 for sample collection information). HIV shedding will be evaluated in relation to the time from the MC surgery to determine whether HIV shedding decreases with wound healing. (Wound healing has been previously characterized in these individuals by a dermascopic examination and assessing keratinization of scar tissue using contact smears on slides and Ayoub-Shaklar staining). In addition, HIV shedding will be evaluated in relation to key characteristics, including HIV plasma viral load and subtype, coital frequency, CD4 cell counts, other viral infection status, and viral suppressive therapy.

**1b. Laboratory methods.** As we have demonstrated in the preliminary results, HIV shedding can be detected using the Amplicor HIV-1 monitor test, version 1.5 ultra-sensitive assay (Roche, Indianapolis, IN). However, it is unknown whether Dacron swabs or lavage provide more consistent results. Thus, we will initially evaluate whether the swabs or lavage of MC wounds provide more consistent results of shedding. Subsequently, we will begin testing all 131 HIV-positive men prior to surgery, at time of MC and then at weekly visits for 8 weeks.

**1c. Expected outcomes and interpretation.** Since MC increased male-to-female HIV transmission by HIV-positive men who engaged in sexual intercourse prior to wound healing [[1](#_ENREF_1)], we expect this aim will demonstrate that HIV viral shedding occurs in healing MC wounds, compared to the preoperative subpreputial samples. We hypothesize that there will be a decrease in viral shedding with progression of wound healing. Shedding within the MC wounds may explain the dichotomy between MC decreasing HIV transmission in observational studies [[2](#_ENREF_2)], but the lack of efficacy in the randomized controlled trial of HIV-positive men and their female partners. We also hypothesize that shedding will correlate with the level of plasma HIV, inversely with CD4 counts, and shedding will decrease with viral treatment.

**1d. Analysis plan.** Statistical analyses will be performed to determine HIV viral shedding from MC wounds compared to preoperative subpreputial shedding. HIV shedding from the surgical wound in HIV-infected men will be determined as a dichotomous variable (HIV RNA detected/not detected) at each study visit. We will compare the proportion of men with detectable HIV and the median time to undetectable shedding between HSV-2-infected and uninfected men. The time to undetectable shedding will be assessed by KM survival analysis and Cox proportional hazards modeling.

The quantity of virus shed among samples with detectable virus will be treated as a continuous variable. Viral shedding will be determined pre-surgery and by time post-surgery, using paired t tests and within-individual changes in HIV viral load over time among HSV-2-positive and HSV-2-negative men. Shedding will be also correlated with plasma HIV viral load and pre-surgical CD4/viremia using linear regression and Pearson correlation coefficients. Changes in viral shedding over time will be modeled using multivariable linear regression.

Univariate analyses will evaluate the association between HIV shedding and sociodemographic characteristics (e.g., age), risk behaviors (e.g., resumption of intercourse, coital frequency and condom use), STI symptoms, HIV viral load, CD4 count, antiretroviral therapy, subtype and stage of infection for HIV-positive individuals. Covariates significantly associated with viral shedding at p <0.15 will be included in the adjusted analyses. Analyses will be conducted using Stata 10.0 (College Station, TX).

**1e. Study power.** Based on our preliminary observation, post-MC HIV wound shedding depends on plasma HIV viral load. Among the 131 HIV-infected men, 116 men have a detectable plasma viral load at time of MC. With 80% power and two-sided alpha=0.05, we will detect that men with detectable plasma viral load are 4.4 times more likely to have HIV wound shedding post-surgery compared to those without detectable HIV plasma viral load. In addition, we expect HIV shedding from MC wounds decrease with wound healing and depends on pre-surgical HIV plasma viral load. Assuming the standard deviation of log10 (plasma viral load) is 0.74 (from our preliminary data), with 80% power and two-sided alpha=0.05, we will detect a hazard ratio of 0.7 with one unit increase in log10 (plasma viral load).

**B. Addendum: Hypotheses and Analysis Plan**

In addition to the two pre-specified hypotheses (i.e., penile HIV shedding correlates with wound healing and penile HIV shedding is associated with plasma viral load) that were included in the original 2010 grant proposal , we investigated the hypothesis that ART suppression of the plasma viral load below detection is associated with significantly decreased penile HIV shedding. We had included ART status in the original grant proposal as a covariate. However, due to the limited number of men originally planned for this study (n=131), we did not think there would be sufficient power to assess this subgroup. Once we were able to include a larger number of men to assess additional study objectives and found that ~10% of HIV-infected men who requested MC also reported ART-use, we decided to investigate this additional hypothesis. Consequently, men were stratified by ART status and detectable viral load. The analytical approaches were as proposed in the original grant submission above.

**Literature Cited**

1. Wawer MJ, Makumbi F, Kigozi G, Serwadda D, Watya S, et al. (2009) Circumcision in HIV-infected men and its effect on HIV transmission to female partners in Rakai, Uganda: a randomised controlled trial. Lancet 374: 229-237.

2. Gray RH, Kiwanuka N, Quinn TC, Sewankambo NK, Serwadda D, et al. (2000) Male circumcision and HIV acquisition and transmission: cohort studies in Rakai, Uganda. Rakai Project Team. Aids 14: 2371-2381.
