# Supplementary material for: HIV Shedding from Male Circumcision Wounds in HIV-Infected Men: A Prospective Cohort Study
Source: PLoS Med. 2015 Apr 28;12(4):e1001820. doi: 10.1371/journal.pmed.1001820 (PMC4412625; doi:10.1371/journal.pmed.1001820)
Supplement: S1 Table — The odds ratios and 95% CIs estimated from the logistic regression models were more extreme than the risk ratios estimated from the Poisson models, as would be expected for common outcomes such as in this study. (DOCX) [file pmed.1001820.s005.docx]

Table S1. Comparison of the adjusted relative risks for Table 2 in the main manuscript using both Poisson and logistic regression models with generalized estimating equations and robust variance estimators showed that the odds ratios and 95%CI were more extreme than the risk ratios estimated from the Poisson models, as would be expected for common outcomes such as in this study.

|  | GEE Poisson | | | | GEE Logistic | | | |
| --- | --- | --- | --- | --- | --- | --- | --- | --- |
|  | Estimate | Lower | Upper | p-val | Estimate | Lower | Upper | p-val |
| ART , plasma VL detected | 0.38 | 0.09 | 1.60 | 0.19 | 0.30 | 0.06 | 1.59 | 0.16 |
| ART , plasma VL suppressed | 0.13 | 0.04 | 0.45 | 0.001 | 0.10 | 0.03 | 0.38 | 0.00 |
| Treatment with co-trimoxazole | 0.87 | 0.55 | 1.37 | 0.55 | 0.84 | 0.47 | 1.51 | 0.56 |
| CD4 <500 and >200 | 1.28 | 0.79 | 2.07 | 0.32 | 1.35 | 0.74 | 2.48 | 0.33 |
| CD4 <200 | 1.91 | 1.12 | 3.27 | 0.018 | 2.43 | 1.17 | 5.06 | 0.02 |
| Wound healed | 0.12 | 0.07 | 0.23 | 0.000 | 0.10 | 0.05 | 0.18 | 0.00 |
| Penile shedding at baseline | 1.96 | 1.21 | 3.19 | 0.007 | 2.63 | 1.21 | 5.69 | 0.01 |
