# Supplementary material for: HIV Shedding from Male Circumcision Wounds in HIV-Infected Men: A Prospective Cohort Study
Source: PLoS Med. 2015 Apr 28;12(4):e1001820. doi: 10.1371/journal.pmed.1001820 (PMC4412625; doi:10.1371/journal.pmed.1001820)
Supplement: S2 Table — (DOCX) [file pmed.1001820.s006.docx]

Table S2. Risk factors for a missed weekly follow-up visit after MC.

*Only assessed among sexually active men.
