# Supplementary material for: HIV Shedding from Male Circumcision Wounds in HIV-Infected Men: A Prospective Cohort Study
Source: PLoS Med. 2015 Apr 28;12(4):e1001820. doi: 10.1371/journal.pmed.1001820 (PMC4412625; doi:10.1371/journal.pmed.1001820)
Supplement: S1 Text — (DOCX) [file pmed.1001820.s007.docx]

Rakai Health Sciences Program Investigators

Heena Brahmbhatt

Larry Chang

Ronald Galiwango

Mary Kate Grabowski

Ronald Gray

Joseph Kagaayi

Caitlin Kennedy

Mohammed Kiddugavu

Godfrey Kigozi

Xiangrong Kong

Oliver Laeyendecker

Tom Lutalo

Fred Makumbi

Gertrude Nakigozi

Fred Nalugoda

Thomas Quinn

Andrew Redd

Steven Reynolds

David Serwadda

Nelson Sewankambo

Robert Sskeubugu

Aaron Tobian

Stephen Watya

Maria Wawer
